# Is standardized care feasible in the emergency setting? A case matched analysis of patients undergoing laparoscopic cholecystectomy

**DOI:** 10.1186/s12893-016-0194-6

**Published:** 2016-12-01

**Authors:** Fabian Grass, Matthieu Cachemaille, Catherine Blanc, Nicolas Fournier, Nermin Halkic, Nicolas Demartines, Martin Hübner

**Affiliations:** 1 Department of Visceral Surgery, University Hospital CHUV, Bugnon 46, 1011 Lausanne, Switzerland; 2 Department of Anaesthesiology, University Hospital CHUV, Lausanne, Switzerland; 3 Institute for Social and Preventive Medicine, University Hospital CHUV, Lausanne, Switzerland

**Keywords:** Cholecystectomy, Emergent, Elective, Postoperative, Pain management

## Abstract

**Background:**

Immediate laparoscopic cholecystectomy is the accepted standard for the treatment of acute cholecystitis. The aim of the present study was to evaluate the feasibility of a standardized approach with tailored care maps for pre- and postoperative care by comparing pain, nausea and patient satisfaction after elective and emergent laparoscopic cholecystectomy.

**Methods:**

From January 2014 until April 2015, data on pain and nausea management were prospectively recorded for all elective and emergency procedures in the department of visceral surgery. This prospective observational study compared consecutive laparoscopic elective *vs.* emergency cholecystectomies. Visual analogue scales (VAS) were used to measure pain, nausea, and satisfaction from recovery room until 96 hours postoperatively.

**Results:**

Final analysis included 168 (79%) elective cholecystectomies and 44 (21%) emergent procedures. Demographics (Age, gender, BMI and ASA-scores) were comparable between the 2 groups. In the emergency group, patients did not receive anxiolytic medication (0% *vs.*13%, *p* = 0.009) and less postoperative nausea and vomiting (PONV) prophylaxis (77% *vs.* 97% *p* = <0.001). Perioperative pain management was similar in terms of opioid consumption (median amount of fentanyl 450ug [IQR 350-500] *vs.* 450ug [375-550], *p* = 0.456) and wound infiltration rates (24% *vs.* 25%, *p* = 0.799). Postoperative consumption of paracetamol, metamizole and opiod medications were similar between the 2 groups. VAS scores for pain (*p* = 0.191) and nausea (*p* = 0.392) were low for both groups. Patient satisfaction was equally high in both clinical settings (VAS 8.5 ± 1.1 *vs.* 8.6 ± 1.1, *p* = 0.68).

**Conclusions:**

A standardized pathway allows equally successful control of pain and nausea after both elective and emergency laparoscopic cholecystectomy.

This study was retrospectively registered by March 01, 2016 in the following trial register: www.researchregistry.com (UIN researchregistry993)

**Electronic supplementary material:**

The online version of this article (doi:10.1186/s12893-016-0194-6) contains supplementary material, which is available to authorized users.

## Background

Cholecystectomy is one of the most commonly performed surgical procedures worldwide [1]. In developed countries, laparoscopic approach is nowadays standard since it has been shown to reduce pain, cosmetic issues, length of stay and morbidity [2]. In the emergency situation of calculous cholecystitis, prompt surgical management has been shown to be equally feasible compared to a *wait and see* approach [3]. To facilitate and harmonize care of patients undergoing laparoscopic cholecystectomy, it might be beneficial to *standardize* both procedure and perioperative management, regardless of the clinical setting. Standardization was achieved by the use of tailored caremaps in the present study, and the surgical procedure was standardized in the setting of our tertiary teaching Institution. Whether such standardization is feasible in both the elective *and* the emergency setting, however, needs to be proven.

The aim of the present study was to compare the perception of nausea and pain and patient satisfaction within the same standardized care pathway after elective and emergent laparoscopic cholecystectomy.

## Methods

### Study design

This is a prospective observational study. Demographic and surgical details and peri- and postoperative pain and nausea management were compared between patients undergoing elective and emergent cholecystectomy. The study cohort included all consecutively operated laparoscopic cholecystectomies between January 2014 and April 2015 at the University Hospital of Lausanne Switzerland (CHUV). Primary open or converted procedures were excluded from this analysis.

Informed written consent regarding this observational study was obtained from all patients before surgery, and the study was approved by the Institutional Review Board (Commission cantonale d'éthique de la recherche sur l'être humain CER-VD). The study was designed according to the STROBE criteria for observational studies and registered under www.researchregistry.com (UIN researchregistry993).

### Surgery and perioperative care

Laparoscopic cholecystectomy was performed in a standardized manner by a classic three- or four-ports approach. The standardized approach was performed or taught by senior staff members of the hepato-biliary unit and all surgeons underwent specific training before performing the procedure by themselves.

Induction of anaesthesia was generally performed with propofol and maintained either by sevoflurane or desflurane or by intravenous propofol in accordance with patients’ pathologies. Rapid sequence induction was performed for all patients suspected to have a full stomach. Opiates (fentanyl or sufentanyl) were administered to each patient intraoperatively associated with paracetamol 1000 mg at the end of the procedure. Non-depolarizing muscle relaxant was administered as needed. As per anaesthetists or surgeons discretion, patients received wound infiltration (bupivacaine 0.25% or naropin 0.25%) or intravenous lidocaine (1.5 mg/kg for induction, then 2 mg/kg/h until recovery room). Postoperative nausea and vomiting (PONV) prophylaxis consisted of administration of dexamethasone 4 mg, droperidol 1 mg and ondansetron 4 mg intraoperatively. Preoperative anxiolytic medication (premedication) was given case by case depending on anaesthesists’ appreciation.

### Data collection

Data was collected prospectively by a study nurse and entered in a dedicated database by two members of the anaesthesiology care team (MC and CB). All patients were treated according to standardized care maps, regardless of the setting (elective or emergent) of the procedure, and no patient was excluded from this analysis, with exception of open or converted cases that were not included at all. In particular, these tailored care-maps for laparoscopic cholecystectomy outlined pre-, peri- and postoperative items such as medications and care protocols for surgeons, anaesthesiologists and nursing staff likewise (Additional file 1).

Baseline demographic (age, gender, body mass index (BMI) and American Society of Anaesthesiologists (ASA) score) and pertinent surgical and anaesthetic information were recorded. These were in particular the mode of surgery (elective *vs.* emergency) and the duration of procedure and anaesthesia. Postoperative consumption of paracetamol, metamizole, tramadol, morphine and oxycodone (delayed-action; Oxycontin® or short-action; Oxynorm®) was assessed and administration of postoperative antiemetic medication (ondansetron, metoclopramide) was recorded. Visual analogue scales (VAS) were used to measure pain (1-10) and patient satisfaction (1-10) from recovery room until 96 hours postoperatively at rest and mobilization. Nausea was assessed and stratified in 4 groups: no nausea = 0, some nausea = 1, occasional (<3/day) vomiting = 2 and frequent (>3/day) vomiting =3. Length of stay (LOS) and in-hospital complications were recorded for both groups.

### Outcomes

The main outcomes of interest were the perception of pain, nausea and satisfaction postoperatively. Demographic and surgical details and peri- and postoperative pain and nausea management were compared between patients undergoing elective *vs.* emergent cholecystectomy.

### Statistical analysis

Data analysis was performed using the Stata Software v. 14.1 (StataCorp, College Station, TX, USA).

Categorical data were summarized as raw frequencies and group percentages. Differences in categorical data distributions between groups were assessed using the chi-squared test, or the Fisher’s exact test in case of insufficient sample size. Confidence intervals for proportions were obtained using the Clopper-Pearson exact method. Continuous data distribution was analyzed using Normal QQ-Plots. Gaussian data were summarized as mean and standard deviation (SD), while non-Gaussian data were summarized as median, interquartile range (IQR) and range. Differences in means between two groups for Gaussian data were assessed using the Student’s t-test. Differences in distribution between two groups for non-Gaussian data were assessed using the Wilcoxon-Mann-Whitney ranksum test. We used a linear mixed-effect model to assess the effect of surgery type on VAS scores, when correcting for time.

A *p*-value < 0.05 was considered statistically significant.

## Results

Two hundred and seventeen patients underwent laparoscopic cholecystectomy during the study period. Three elective (2%) and 2 emergency (4%) interventions needed pre-emptive conversion to open approach for technical reasons and were excluded, leaving 212 patients for final analysis. One hundred and sixty-eight (79%) elective cholecystectomies were compared to 44 (21%) emergent procedures. Acute cholecystitis was the indication for 32 emergent procedures (73%) with the remaining (27%) being symptomatic gallstone disease. Demographics (Age, gender, BMI and ASA-scores) are outlined in Table 1. After propensity score calculation, no differences were found between these 4 parameters, and for this reason case matching was not necessary and not performed. Intraoperative pain management (amounts of opioids and fentanyl, wound infiltration rates) did not differ between the 2 groups, as demonstrated in Table 2.

**Table 1:** Demographics

	Elective (*n* = 168)	Emergency (*n* = 44)	All patients (*n* = 212)	*P*
Age [years]				
(median, IQR)	52, 37-71	56, 40-69	53, 38-70	0.667
Gender				
Female (%) Male (%)	92 (54.8) 76 (45.2)	27 (61.4) 17 (38.6)	119 (56.1) 93 (43.9)	0.432
BMI [kg/m2]				
(median, IQR)	27, 24-30	26, 23-29	27, 24-30	0.145
ASA group				
I II III	24 (14.3) 125 (74.4) 19 (11.3)	6 (13.6) 33 (75.0) 5 (11.4)	30 (14.2) 158 (74.5) 22 (11.3)	0.994
Duration of procedure [min]				
(median, IQR)	100, 85-122	104, 87-123	101, 86-123	0.483
Duration of anesthesia [min]				
(median, IQR)	131, 114-154	136, 115-150	131, 114-153	0.767

Comparison of baseline characteristics by comparing patients who underwent elective cholecystectomy with patients who underwent emergency cholecystectomy

*BMI* Body Mass Index, *ASA* American Society of Anesthesiologists

**Table 2:** Intraoperative pain management

	Elective (*n* = 168)	Emergency (*n* = 44)	All patients (*n* = 212)	*P*
**Type of anesthesia**				
Gas IV	155 (92.3) 13 (7.7)	39 (90.7) 4 (9.3)	194 (91.9) 17 (8.1)	0.755
**IV lidocaïne**	16 (9.5)	1 (2.3)	17 (8)	0.207
**Wound infiltration**	41 (24.4)	11 (25)	52 (24.5)	0.935
- **amount [mL]**				
(median, IQR)	20, 20-40	20, 20-40	20, 20-40	0.799
**Administration of opiates**	168 (100)	44 (100)	212 (100)	
- **fentanyl [μg]**				
(median, IQR)	450, 350-500	450, 375-550	450, 350-500	0.456
- **sufentanyl [μg]**				
(median, IQR) **Premedication**	36, 35-40 21 (12.5)	26, 25-40 0 (0)	35, 25-40 21 (9.9)	0.178 0.009
**PONV prophylaxis**	163 (97)	34 (77.3)	197 (92.9)	**< 0.001**

Comparison of intraoperative pain management by comparing elective cholecystectomies with emergency cholecystectomies

*IV* intravenous, *PONV* Postoperative Nausea and Vomiting

Bold characters indicate significant values (*p* < 0.05)

In emergency situation, patients did not receive anxiolytic medication (0% *vs.*13%, *p* = 0.009) and less postoperative nausea and vomiting (PONV) prophylaxis (77% *vs.* 97%, *p* = <0.001) (Table 2).

**Fig. 1:**
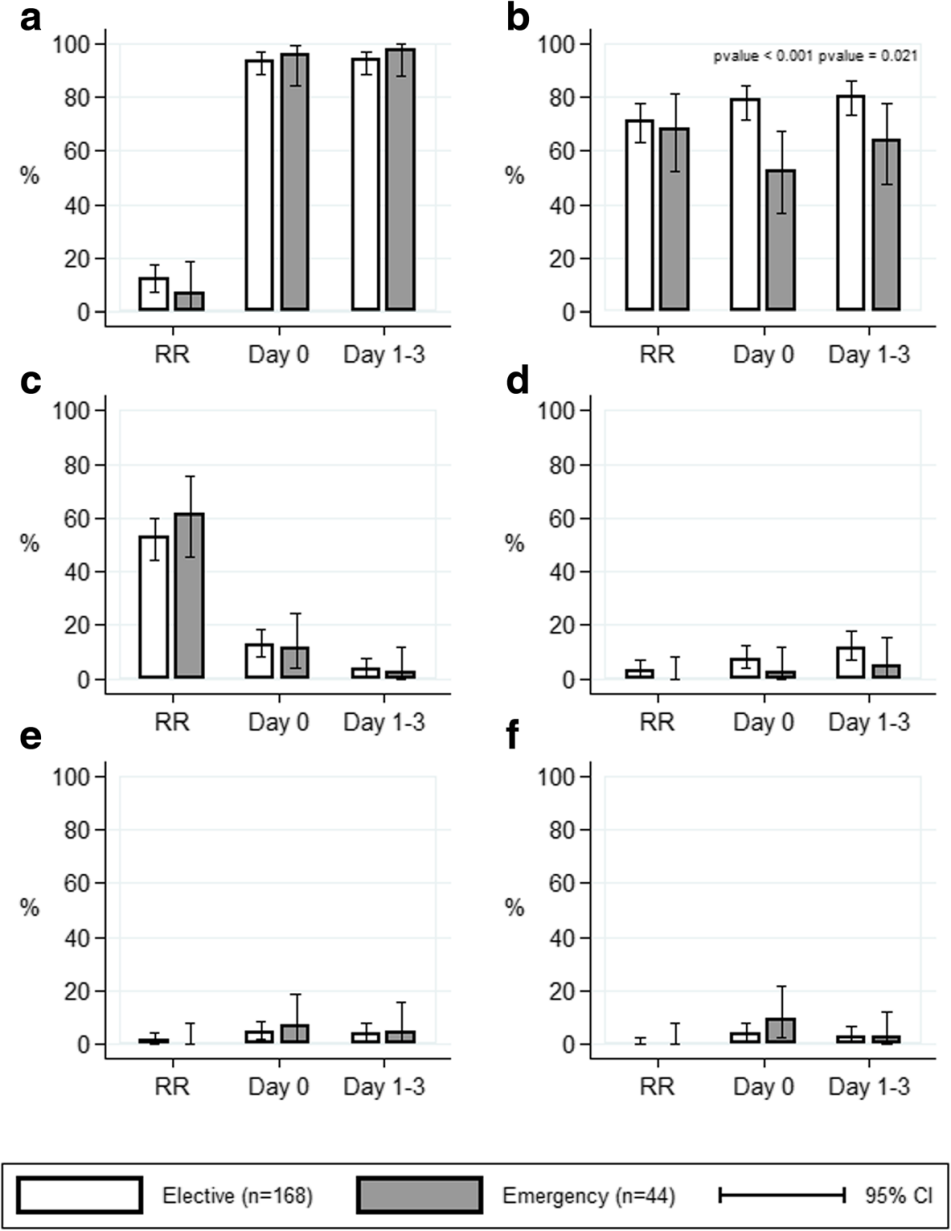
Postoperative pain management. Comparison of proportion of patients receiving postoperative pain medication (subgraphs) at different time points between patients who underwent elective cholecystectomy (white bars) and patients who underwent emergency cholecystectomy (grey bars). For readability purpose, p values of > 0.05 not displayed. Subgraphs: **a**) Paracetamol **b**) Metamizole **c**) IV/SC morphium **d**) Tramadol **e**) Oxycodone (Oxynorm®) **f**) Oxycodone (Oxycontin®). RR – recovery room, IV – intravenous, SC – subcutaneous

**Fig. 2:**
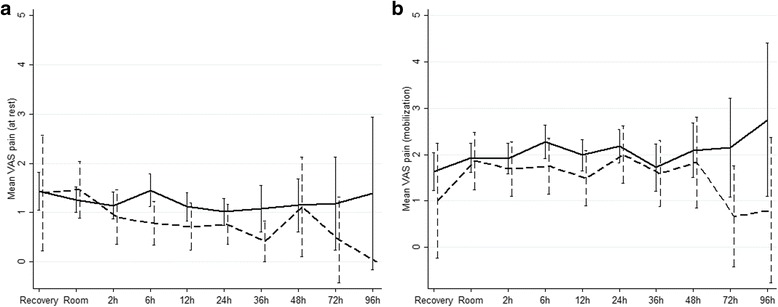
Comparison of VAS scores. Comparison of VAS scores for pain at different time points postoperatively by comparing elective (*n* = 168, continuous line) and emergent (*n* = 44, dashed line) cholecystectomy. **a**) at rest (*p = 0.191*, linear mixed model adjusted for time). **b**) at mobilization (*p = 0.16*, linear mixed model adjusted for time). VAS – Visual Analogue Scale

Postoperative consumption of paracetamol, metamizole and opioid medications were similar between the 2 groups, as illustrated in Fig. 1. VAS scores for pain at rest and under mobilization did not show significant differences, as shown in Fig. 2. Nausea was rarely an issue in both groups (Score ≥ 1 at 6 h: 7% *vs.* 6%, *p* = 0.362). Postoperative antiemetic medication was rarely administered in both groups (18% *vs.* 14%, *p* = 0.507). Patient satisfaction was equally high in both groups (VAS 8.5 ± 1.1 *vs.* 8.6 ± 1.1, *p* = 0.68).

Six in-hospital complications (4%) were recorded in the elective group. One patient presented with a postoperative bile leak and needed endoscopic retrograde cholangiopancreatography at postoperative day (POD) 12. Another patient presented with postoperative bleeding and needed arterial embolization at POD 4. The 4 remaining complications were general issues: one acute myocardial infarction, one pneumonia, one allergic reaction to metamizole and one case of postoperative nausea and vomiting (PONV). After emergency cholecystectomy, one patient (2%, *p* = 0.668) presented with postoperative bleeding, needing laparoscopic re-intervention.

Median length of stay was 1 day [0-19] in the elective and 2 days [0-8] in the emergency group (*p* = 0.072).

## Discussion

Pain management was similar after elective and emergent laparoscopic cholecystectomy. Consecutively, postoperative pain and nausea scores were similar and patients’ satisfaction equally high. *One single* standardized pathway appears to fit for laparoscopic cholecystectomy in the elective but also in the emergency situation.

Standardized care maps for a specific procedure might be a way to facilitate perioperative management by standardising and ease pre-, intra- and postoperative care [4]. This standardization aims to simplify work for caregivers and might be especially useful in frequently performed surgical procedures like laparoscopic cholecystectomy. The present study aimed to assess patients’ postoperative perception of pain and nausea within a standardized pathway comparing elective and emergent laparoscopic cholecystectomies. Few differences were observed in patient preparation and perioperative pain management, and outcome in terms of pain control and perception of nausea were equally encouraging in both settings. These findings emphasize the utility of care maps even in emergency situation for one of the most commonly performed procedures, resulting in high patient satisfaction.

Pain is obviously an important issue for the surgical patient and adequate management of major concern since it might delay patient discharge [5–7]. Several manifestations of laparoscopic cholecystectomy related postoperative pain were described: Visceral pain related to tissue injury and stretching of nerve endings, parietal pain related to port sites and referral shoulder pain related to stretching and irritation of the diaphragm by carbon dioxide gas [8, 9]. Ways to minimize postoperative pain might thus be a restricted number and size of ports [10] or the avoidance of residual pneumoperitoneum at the end of the procedure [9].

Several pain management strategies have been investigated for laparoscopic cholecystectomy. A recent randomized controlled trial showed a benefit for intravenous lidocaine infusion by reducing postoperative pain and opioid consumption [11]. Wound infiltration by local anaesthetics is safe and might add some reduction in pain. However, a recent meta-analysis concluded that the quality of evidence was very low and the clinical importance small [12]. An alternative might be Transversus Abdominis Plane (TAP) block as an adjunct to multimodal postoperative analgesia [13]. In the present study, only a small proportion of patients received local anesthetics either by intravenous or local administration. Consequently, comparison of these specific interventional techniques was not performed.

Even if conversion rates are two- to three-fold higher in emergently performed cholecystectomies, a recent study showed no significant difference in morbidity or mortality, supporting early surgical management in emergency situations [14]. These findings have been confirmed by a recently performed randomized clinical trial of our group, which showed that prompt surgical management of acute cholecystitis is feasible regardless of the onset of symptoms (Roulin et al., 2016, *Ann Surg, in press*). However, increased length of hospital stay has been described after emergent cholecystectomy, whereas discharge at the first postoperative day seemed realistic without negative impact on outcomes [15]. Median hospital stay was low in both groups in the present study. Patients stayed one more day after emergent cholecystectomy, assumingly to extend the observation period in the context of emergency, but these patients did not experience more pain or nausea. Of note, the duration of the procedure was rather long in both groups, a finding that might be explained by the teaching tasks of our academic institution. In fact all cholecystectomies were performed by residents under supervision by a staff surgeon.

One reason for these positive results might be the use of care maps, with standardized, peri- and postoperative patient care regardless of the elective or emergent setting. The beneficial effect of standardization has been repeatedly shown within Enhanced Revovery After Surgery (ERAS) pathways [16], coming along with decreased nursing workload [17] and increased patient and provider satisfaction [4]. Further, besides clinical benefits, economically relevant benefits for the utilization of standardised clinical pathways with reduction in use of resources have been described [18].

Several limitations of this study need to be addressed. The cohort is small, and the analysis was performed retrospectively. Confirmation of our findings by independent groups is therefore necessary.

## Conclusions

A standardised pathway allowed equally successful control of pain and nausea after elective and emergency cholecystectomy resulting in high patient satisfaction in both situations.

## Additional file

Additional file 1: Institutional standardized care map for laparoscopic cholecystectomy (DOCX 42 kb)

## Abbreviations

BMI: Body mass index
PONV: Postoperative nausea and vomiting
