# Supplementary material for: Is standardized care feasible in the emergency setting? A case matched analysis of patients undergoing laparoscopic cholecystectomy
Source: BMC Surg. 2016 Dec 1;16:78. doi: 10.1186/s12893-016-0194-6 (PMC5131530; doi:10.1186/s12893-016-0194-6)
Supplement: Additional file 1: — Institutional standardized care map for laparoscopic cholecystectomy (DOCX 42 kb) [file 12893_2016_194_MOESM1_ESM.docx]

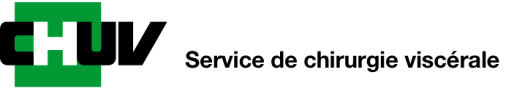
 Caremap Laparoscopic cholecystectomy

|  | **Pre-hosp surgical/anesthesiologic consultation** | **d -1** | **d0 (surgery)** |
| --- | --- | --- | --- |
| INTERVENANTS |  |  |  |
| Surgeon | Indication, information  Consent  Schedule  Consultation report  Patient history + exam performed by resident | Ward round  Entry exam  Patient check mark  Verification : Medical chartRadiologic chartLabConsent | Surgery Postoperative ward round |
| Anesthesiologist | Anesthesiologic evaluation  Consent | Ward round  Anesthetic consent |  |
| Nurse outpatient clinic | Outpatient chart  Nurse and medical chart  Digital forms :  Contacts and intervenants  General information  Vital signs |  |  |

| PARACLINIC EXAMS | |  |  |
| --- | --- | --- | --- |
| Radiology | According to anesthesiologist |  |  |
| Lab | According to anesthesiologist Blood group | Type & Screen |  |
| Other | ECG according to anesthesiologist |  |  |

| **MEDICATION** |  |  |  |
| --- | --- | --- | --- |
| Personal medication | Personal medication  Stop anticoagulation at d-x | Personal medication  (besides anticoagulation)  Verification anticoagulation stopped | According to anesthesiologist |
| Premedication |  |  | According to anesthesiologist |
| Sedation |  | According to anesthesiologist | Stilnox 10 mg 1x/d PO ®  (if < 75 y) |
| Anti-infectious prophylaxis |  |  |  |
| Perfusion |  |  | Glucosalin 1/3-2/3 1000 mL/24h IV |
| Antithrombotic prophylaxis |  | **Clexane 40 mg 1x/d SC** | Clexane 40 mg 1x/d SC12h post-op. |
| Pain management |  |  | Paracetamol 1 g 4x/d IV **Metamizole 500 mg 4x/d IV**  Morphine 0,1 mg/kg 6x/d SC ® |
| Antiemetic medication |  |  | Metoclopramide 10 mg 3x/d IV ®  Odansetron 4 mg 3x/d IV ® |

| NUTRITION |  |  |  |
| --- | --- | --- | --- |
| Oral alimentation |  | Normal  NPO from midnight | Pre-op. : NPO  Post-op. : Drinks when completely awake  Normal 6h after |

| NURSE CARE |  |  |  |
| --- | --- | --- | --- |
| Vital signs | 1x | 1x at admission | Pre-op. : 1x/d  Post-op. 3x/d |
| Weight |  | 1x at admission |  |
| Mobilisation |  | Free | First mobilisation under supervision |
| Surveillances |  | Reception, installation  Evaluate inquietude  Evaluate allergies  Pre-op shower | Pre-op. : Pre-op shower  Post-op. : Equipment, pain, urine output, transit, dressing |
| Material |  |  | Venflon |
| Education |  | Explain process of hospitalisation,  1st post-op mobilisation | Reminder of pre-op explanations |
| Administration |  | Verification :  Medical chart  Nurse chart Digital forms : Entry nurse, General settings, contacts and intervenants, Braden score | Verification :  Institutional security form signed and documented  Operating room departure time scheduled |

**Bold characters : imperative prescriptions**

Slim characters : optional prescriptions = ®


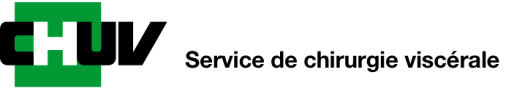


Caremap Laparoscopic cholecystectomy

|  | **d1** | **d2 (departure)** |
| --- | --- | --- |
| INTERVENANTS |  |  |
| Surgeon | Ward round | Ward round  Organisation departure  Prescriptions  Sick leave  FAX for treating general practitioner  Outpatient schedule:  Removal of stitches at d10  Final control at 1 month |
| Anesthesiologist |  |  |
| Nurse outpatient clinic |  |  |

| **PARACLINIC EXAMS** | |  |
| --- | --- | --- |
| Radiology |  |  |
| Lab |  |  |
| Other |  |  |

| **MEDICATION** |  |  |
| --- | --- | --- |
| **Personal medication** | Personal medication  Anticoagulation restarted | Personal medication |
| **Premedication** |  |  |
| **Sedation** | Stilnox 10 mg 1x/d PO ®  (if < 75 y) |  |
| **Perfusion** |  |  |
| **Antithrombotic prophylaxis** | Clexane 40 mg 1x/j SC |  |
| **Pain management** | Paracetamol 1 g 4x/d PO  Metamizole 500 mg 4x/d PO  Morphine 0,1mg/kg 6x/d SC ® | Paracetamol 1 g 4x/j PO  Metamizole 500 mg 4x/j PO |
| **Antiemetic medication** | Metoclopramide 10 mg 3x/j IVD ®  Ondansetron 4 mg 3x/j IV ® |  |

| NUTRITION |  |  |
| --- | --- | --- |
| Oral alimentation | Normal | Normal |

| NURSE CARE |  |  |
| --- | --- | --- |
| Vital signs | 3x/j | 1x/j |
| Mobilisation | Free | Free |
| Surveillances | Equipment, pain, transit, dressing | Equipment, pain, dressing  Removal : equipment. dressing |
| Material | Venflon | Removal venflon |
| Education |  | Departure advice :  Wound care, stitches, cough, bath/shower, sports, sun |
| Administration |  | Departure :  Handover prescriptions  Sick leave  Appointments  Digital forms :  Departure nurset  Closure of chart |

**Bold characters : imperative prescriptions**

Slim characters : optional prescriptions = ®
